# Current profile of Charcot‐Marie‐Tooth disease in Africa: A systematic review

**DOI:** 10.1111/jns.12489

**Published:** 2022-04-05

**Authors:** Abdoulaye Yalcouyé, Kevin Esoh, Landouré Guida, Ambroise Wonkam

**Affiliations:** ^1^ Faculté de Médecine et d'Odontostomatologie USTTB Bamako Mali; ^2^ Division of Human Genetics, Department of Pathology, Faculty of Health Sciences University of Cape Town Cape Town South Africa; ^3^ Neurogenetics Branch National Institute of Neurological Disorders and Stroke Bethesda Maryland USA; ^4^ Service de Neurologie Centre Hospitalier Universitaire du Point “G” Bamako Mali; ^5^ McKusick‐Nathans Institute, and Department of Genetic Medicine Johns Hopkins University School of Medicine Baltimore Maryland USA

**Keywords:** Africa, Charcot‐Marie‐Tooth disease, clinical, epidemiology, genetics

## Abstract

**Background and aims:**

Charcot‐Marie‐Tooth disease (CMT) is the most common inherited peripheral neuropathy characterised by a high clinical and genetic heterogeneity. While most cases were described in populations with Caucasian ancestry, genetic research on CMT in Africa is scant. Only a few cases of CMT have been reported, mainly from North Africa. The current study aimed to summarise available data on CMT in Africa, with emphasis on the epidemiological, clinical, and genetic features.

**Methods:**

We searched PubMed, Scopus, Web of Sciences, and the African Journal Online for articles published from the database inception until April 2021 using specific keywords. A total of 398 articles were screened, and 28 fulfilled our selection criteria.

**Results:**

A total of 107 families totalling 185 patients were reported. Most studies were reported from North Africa (n = 22). The demyelinating form of CMT was the commonest subtype, and the phenotype varied greatly between families, and one family (1%) of CMT associated with hearing impairment was reported. The inheritance pattern was autosomal recessive in 91.2% (n = 97/107) of families. CMT‐associated variants were reported in 11 genes: *LMNA*, *GDAP1*, *GJB1*, *MPZ*, *MTMR13*, *MTMR2*, *PRX*, *FGD4/FRABIN*, *PMP22*, *SH3TC2*, and *GARS*. The most common genes reported are *LMNA*, *GDAP1*, and *SH3TC2* and have been found mostly in Northern African populations.

**Interpretation:**

This study reveals that CMT is not rare in Africa, and describes the current clinical and genetic profile. The review emphasised the urgent need to invest in genetic research to inform counselling, prevention, and care for CMT in numerous settings on the continent.

## 1 INTRODUCTION

An estimated 2%‐7% of the world population suffers from peripheral neuropathy (PN).^1^ Although rarely life‐threatening, PN can be severely disabling, leading to wheelchair dependence. PN can be of either genetic or non‐genetic aetiology. While symptomatic and curative treatments for PNs are possible via addressing the underlying aetiology, with subsequent nerve cell regeneration and resolution of the condition, curative treatments for PNs of genetic origin have been elusive.^2^

Charcot‐Marie‐Tooth disease (CMT) also known as hereditary motor and sensory neuropathy, is the most common PN group with a high clinical and genetic heterogeneity. Population‐based studies have reported variable prevalence,^3^ with a crude global estimate of 1/2500.^4^ Yet, despite being described more than 130 years ago and the genetic cause identified about 30 years ago,^5^, ^6^ there remains a paucity of information on its global prevalence and genetic epidemiology due largely to challenges in diagnosis, especially in countries with limited resources. Studies of CMT in Africa, particularly genetic epidemiology are notably scarce,^7^ because of the unavailability of neurologists and diagnostic tools.^8^

Classically, CMT is divided into two main types: type 1 (CMT1) when the disease is primarily demyelinating with the median motor nerve conduction velocities (MNCVs) <38 m/s, and type 2 (CMT2) when the disease is axonal with MNCVs >38 m/s.^9^ An intermediate type is suggested when the MNCVs are between 25 and 45 m/s. Other sub‐classifications are based on the inheritance pattern, which can be autosomal dominant (AD), autosomal recessive (AR), or X‐linked (CMTX).^9^, ^10^

Over 100 genes have been associated with CMT,^9^ and it is reported that over 90% of all genetically diagnosed cases are due to mutations in four genes: *PMP22*, *GJB1*, *MFN2*, and *MPZ*.^9^, ^10^ The 1.4 Mb duplication on chromosome 17 (17p) accounts for over 60% of all genetically diagnosed cases of CMT in Europe and America.^9^ This region contains nine genes including the peripheral myelin protein 22 gene (*PMP22*), which is emendable to therapeutic manipulation of CMT1A, mainly aiming at reducing *PMP22* transcription.^2^ Still, no curative treatment exists for these CMTs, although several clinical trials are ongoing.^11^, ^12^, ^13^

Interestingly, *PMP22* has been associated with CMT in only four families in Africa.^14^, ^15^, ^16^ This may be due to the limited studies on CMT in the continent or, alternatively, the genetic architecture of CMT among people of African and non‐African ancestries is significantly different, as demonstrated with a higher proportion of CMT4B in Tunisia.^15^, ^16^ Substantial genetic architectural differences among people of African and non‐African ancestries have been extensively documented,^14^, ^17^, ^18^ and the highest genetic diversity among Africans should be expected to be associated with numerous unreported variants in known genes, and offer the opportunity for novel genes discovery, as shown in congenital hearing impairment research.^19^, ^20^ Hence, current therapeutic strategies under trial may not be beneficial to Africans, unless the relevant genetic variants for these populations are fully identified.^21^

Therefore, given the extensive genetic diversity in Africa,^22^ the high consanguinity in numerous regions^23^, ^24^ and fertility rates,^25^ African populations present a unique opportunity to discover novel disease variants^25^ and, specifically to better understand CMT pathophysiological mechanisms.^26^ In this review, we report the scarcity of research on CMT in Africa, the current clinical profiles, the specificity in the pattern of inheritance, and available genetic data of reported CMT cases in Africa.

## 2 METHODS

The present review was performed in accordance with the guidelines for transparent reporting of systematic reviews and meta‐analyses (PRISMA statement 2020).

### 2.1 Search strategy

We searched four databases for articles reporting CMT in Africa that fitted with the aim of this study. These databases included PubMed (https://pubmed.ncbi.nlm.nih.gov), Scopus (https://www.scpus.com), African Journals Online (https://ajol.info), and Web of Sciences (https://clarivate.com/products/web-of-science/). We used the following keywords: (“Charcot Marie Tooth disease” OR CMT OR “hereditary motor and sensory neuropathy”) AND Africa. The structured search strategy was designed to identify any published articles that report epidemiological, clinical, and genetic studies of CMT in Africa. Articles published in both English and French were included.

### 2.2 Selection criteria

We included observational studies published from database inception until April 2021 that report data on the epidemiology, clinical, and genetic features of CMT in Africa. In case of duplicate studies, we selected the most recent or more informative studies. We excluded qualitative studies, letters to the editor, reviews, and commentaries. Also, studies with unavailable full text or missing key data were removed from this review. In the case of articles reporting on patients both from Africa and outside Africa, we extracted the data of interest.

### 2.3 Selection of studies

All titles, abstracts, and full‐text articles were independently screened by two reviewers (A.Y. and K.E.). All these articles were physically downloaded and imported into Endnote version X9.1 (Bld 12 691). One author (A.Y.) analysed the articles before submitting them to the second author (K.E.) to cross‐check the accuracy. Any disagreements between the two authors were solved by consensus and discussion.

### 2.4 Data extraction process and assessment of methodological quality

One researcher (A.Y.) extracted data from the studies included in this review. A second researcher (K.E.) checked the accuracy of the extraction process. Any discrepancy was resolved through discussion and consensus. We extracted data included the last name of the first author, the year of publication, the country of origin of the patients, the prevalence, the incidence, the study setting, the study design, the sex ratio, age ranges, the sample size, the number of affected individuals, type of CMT, the age onset, the starting symptoms, the major neurological signs, the inheritance pattern, the technique used to identify genes, the identified gene or/and variants, and the reporting journal. We also extracted data from the available histological studies.

The two investigators (K.E. and A.Y.) assessed the risk of bias and the quality of included studies using the quality of genetic studies (Q‐Genie) tool developed by Sohani et al^27^ for genetic studies and the risk of a bias assessment tool for prevalence studies developed by Hoy et al^28^ for the other studies. Discrepancies were solved by discussion and consensus.

## 3 RESULTS

**FIGURE 1:**
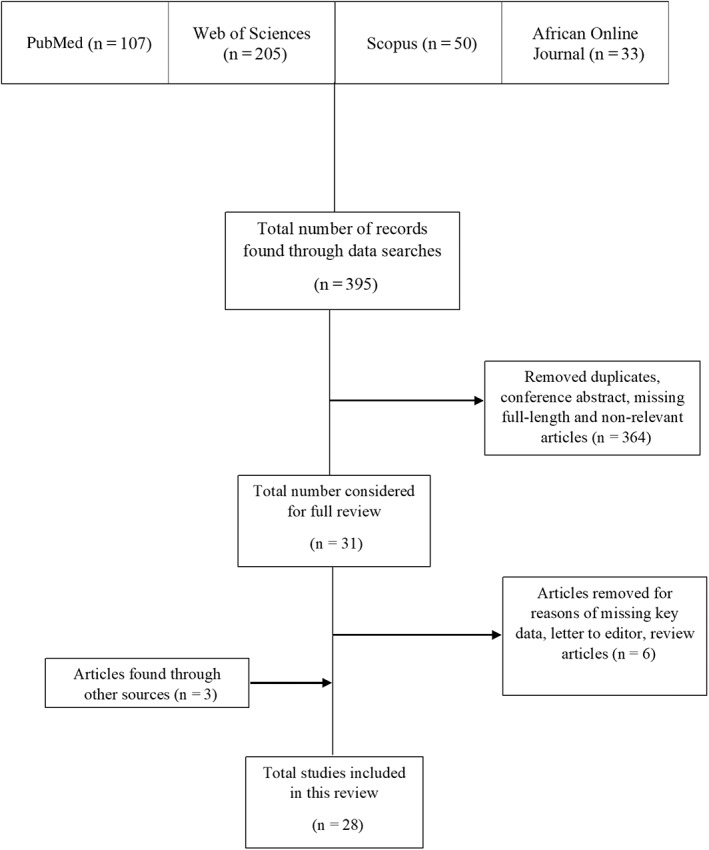
Flow chart of studies selection

An initial 395 records were identified. We removed 364 articles after screening for titles and abstracts. The remaining 31 records were considered for the full review, after which we removed six records for reasons of missing key data, the letter to the editor, or review articles. In addition, three articles were found through other sources. Finally, a total of 28 articles fulfilled our selection criteria and were included in the review (Figure 1).

### 3.1 Epidemiology of CMT

**FIGURE 2:**
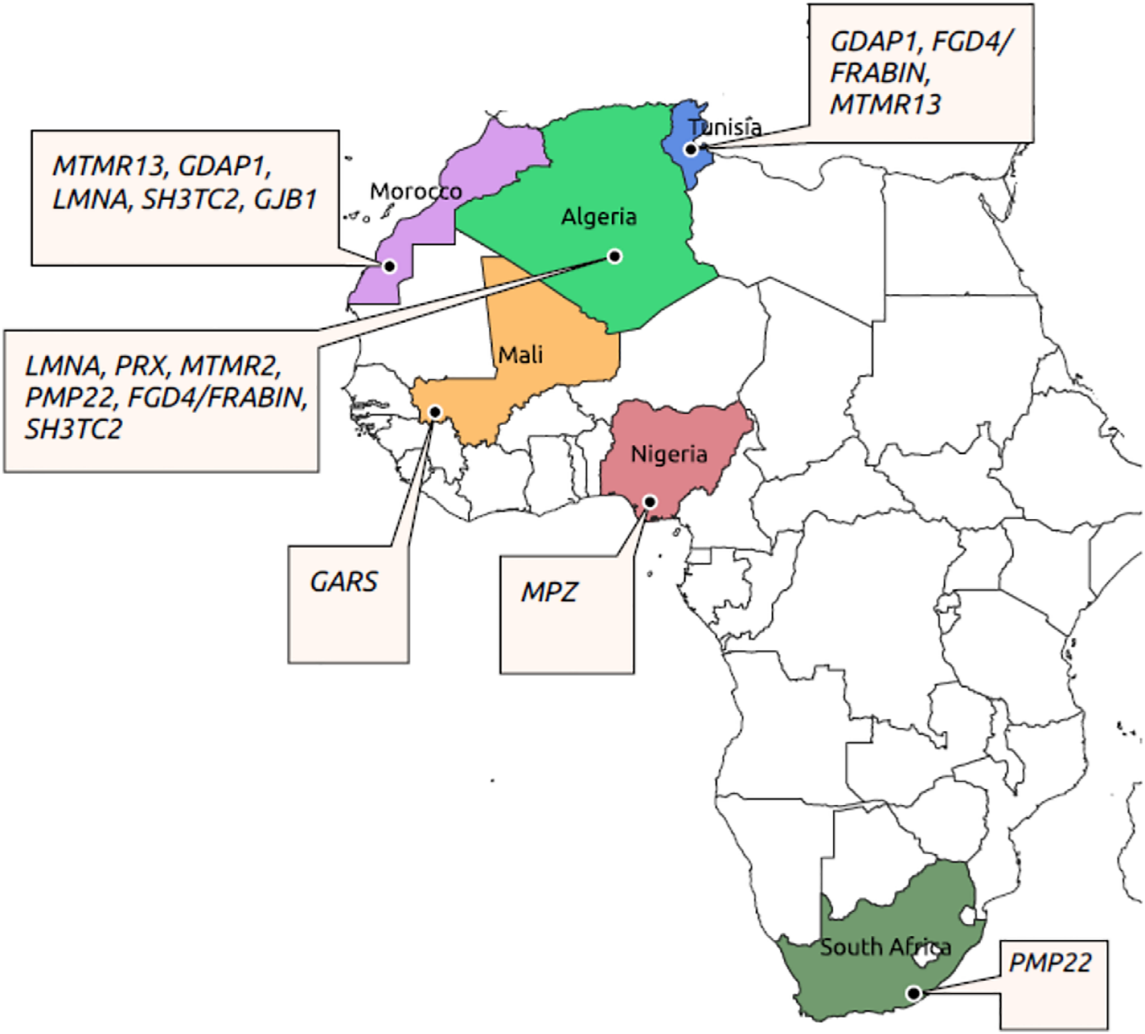
Genes reported in respective African countries

Of the 28 studies included in this review, only one was population‐based, namely a community‐based study from Egypt that included 42.223 individuals, five patients were found with CMT phenotypes, representing an estimated prevalence of 12/100.000.^29^ One hospital‐based and cross‐sectional study in Nigeria reported a frequency of 0.15/100.000 among 2.1 million patients seen for neurodegenerative diseases.^30^ Most of the studies were case reports, and only seven were cross‐sectional studies. The total number of affected individuals per study varied from one to 42 patients. The age at diagnosis ranged from 4 to 70 years (22/28 studies), and was not specified in six studies. Most of the studies (78.6%; n = 22/28) were reported from Northern Africa, including Algeria, Tunisia, Morocco, and Egypt, and only 21.4% (n = 28) in sub‐Saharan Africa (SSA) including one from Mali, two from South Africa and three from Nigeria. All the descriptive features are summarised in Table 1 and Figure 2.

**TABLE 1:** Epidemiological aspects of the studies included in this review

First author's name, publication year	Study design	Study setting	Incidence	Prevalence	Sample size	Number of affected	Age range	Proportion of male (%)	Reporting journal
Aiyesimoju, 1984^30^	Cross sectional study	Hospital	NR	0.15/100000^F^	2.1^M^	3	28‐43	66.7 (n = 3)	*Neurology*
LeGuern, 1996^67^	Case report	Hospital	NR	NR	2^a^	11	NR	54.5 (n = 11)	*Human Molecular Genetics*
Kessali, 1997^44^	Case report	Hospital	NR	NR	25	12	11‐28	58.3 (n = 12)	*Neurology*
Meggouh, 1998^43^	Case report	Hospital	NR	NR	6	1	17	0	*Journal of Medical Genetics*
Bouhouche, 1999^39^	Case report	Hospital	NR	NR	17	9	15‐49	89 (n = 9)	*American Journal of Human Genetics*
Othmane, 1999^16^	Case report	Hospital	NR	NR	26	9	NR	NR	*Genomics*
Barhoumi, 2001^38^	Case report	Hospital	NR	NR	24	13	19‐70	7.7 (n = 13)	*Neuromuscular Disorders*
Baxter, 2001^33^	Case report	hospital	NR	NR	4^a^	8	NR	NR	*Nature Genetics*
Sandre‐Giovannoli, 2002^68^	Cross sectional study	Hospital	NR	NR	23^a^	NR	NR	NR	*American Journal of Human Genetics*
Kakar, 2003^35^	Case report	Hospital	NR	NR	7	1	72	100 (n = 1)	*Muscle and Nerve*
Azzedine, 2003^31^	Case report	Hospital	NR	NR	30	7	NR	14.3 (n = 7)	*American Journal of Human Genetics*
Chaouch, 2003^45^	Case report	Hospital	NR	NR	4^a^	8	16‐30	62.5 (n = 8)	*Neuromuscular Disorders*
Birouk, 2003^23^	Case report	Hospital	NR	NR	17	4	15‐20	0	*Arch Neurology*
Tazir, 2004^42^	Cross sectional study	Hospital	NR	NR	62	21	12‐45	62 (n = 21)	*Brain*
Azzedine, 2006^69^	Cross sectional study	NS	NR	NR	4^a^	NS	NS	NS	*Neurology*
Bösenberg, 2006^48^	Case report	Hospital	NR	NR	2	2	14‐19	100 (n = 2)	*Southern African Journal of Anaesthesia & Analgesia*
Onwuewe, 2007^50^	Case report	Hospital	NR	NR	1	1	31	100 (n = 1)	*Journal of College of Medicine*
Bouhouche, 2007^41^	Case report	Hospital	NR	NR	11	6	4‐19	50 (n = 6)	*Canadian Journal of Neurological Sciences*
Delague, 2007^70^	Case report	Hospital	NR	NR	7	3	NR	100 (n = 3)	*American Journal of Human Genetics*
Bouhouche, 2007^40^	Cross sectional study	Hospital	NR	NR	95	31	4‐49	42 (n = 31)	*Brain*
Hamadouche, 2008^49^	Cross sectional study	Hospital	NR	NR	25^a^	42	NR	48 (n = 42)	*Annals of Human Genetics*
Nouioua, 2011^37^	Cross sectional study	Hospital	NR	NR	2^a^	7	9‐22	85.7 (n = 7)	*Neuromuscular Disorders*
Baudot, 2012^32^	Case report	Hospital	NR	NR	1^a^	1	16	100 (n = 1)	*Journal of the Peripheral Nervous System*
Kandil, 2012^29^	Cross sectional study	Community	NR	1.2/10.000	42.223	5	NR	80 (n = 4)	*Neurological Research*
Boubaker, 2013^34^	Case report	Hospital	NR	NR	8	3	6‐22	33.3 (n = 3)	*Annals of Human Genetics*
Mathis, 2014^36^	Case report	Hospital	NR	NR	1	1	10	0	*Neuromuscular Disorders*
Yalcouyé, 2019^7^	Case report	Hospital	NR	NR	4	3	37 (19‐58)	33 (n = 1)	*Molecular Genetics and Genomic Medicine*
Manyeruke, 2020^46^	Case report	Hospital	NR	NR	1	1	11	0	*South African Ophthalmology Journal*

*Note*: Superscript F indicates hospital frequency and superscript M indicates million.

Abbreviations: NR, not reported; NS, not specified.

^a^ Number of families.

### 3.2 Clinical expression

**FIGURE 3:**
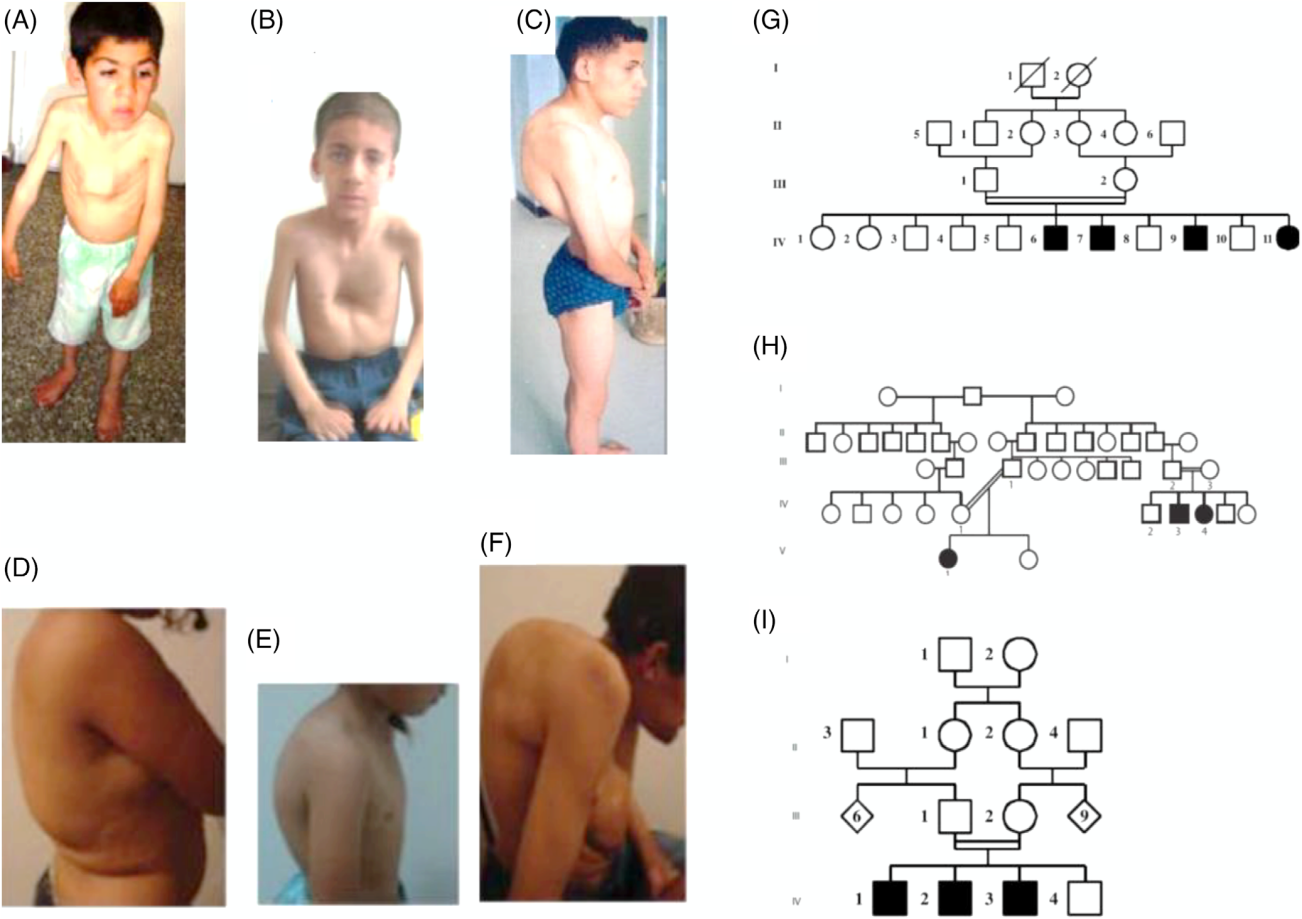
Illustration of some phenotypes of CMTs. A‐C, Images of Charcot‐Marie‐Tooth disease (CMT) patients with mutation in *MTMR2* genes showing the chest deformities, dyspnoea, and severe scoliosis. D‐F, Images of CMT patients with mutation in *FGD4/FRABIN* gene showing a severe kyphoscoliosis. G‐I, Pedigrees of some families showing autosomal recessive transmission manner with the consanguinity (images extracted from the articles by Yalcouyé et al)^7^, ^34^, ^37^

A total of 185 patients were described in the studies reviewed, and the sex ratio was 1.2 (99 males vs 86 females). The demyelinating CMT type was reported in 58.3% of the studies,^16^, ^31^, ^32^, ^33^, ^34^, ^35^, ^36^, ^37^ followed by the axonal type in 37.5% (n = 28)^23^, ^38^, ^39^, ^40^, ^41^, ^42^ and the intermediate form in 4.2%.^43^ The disease started mostly in the first two decades of life but cases with later onset were also reported.^35^ Almost all studies reported muscle weakness predominantly in the lower limbs as the starting symptoms, and only few cases reported sensory impairment as presenting symptoms.^7^, ^23^, ^31^, ^34^, ^35^, ^36^, ^40^, ^42^, ^44^ The major neurological signs included muscle weakness and wasting, predominantly in distal limbs but proximal involvement was reported in some studies.^39^, ^45^ In addition, other neurological signs such as steppage gait, skeletal deformities (*pes cavus* and *pes planus*, hammer toes, claw hands, scoliosis, and kyphosis) (Figure 3C‐F), and sensory impairment were reported.^34^ Reflexes were reduced to absent in most of the cases. However, a case with upper motor neuron involvement with brisk reflexes was reported.^38^ A severe case associated with marked stridor during inspiration causing dyspnoea and abdominal respiration, and a vocal cord paralysis was reported in a family with three affected sibs^37^ (Figure 3A‐C).

A rare case of the AR demyelinating form of CMT associated with early‐onset glaucoma was reported from Tunisia.^31^ Moreover, an unusual phenotype associating CMT1A with macular oedema was first reported in a South African girl.^46^ Only one sporadic case of intermediate motor NCV in a female from Morocco was reported with a mutation in the *GJB1* gene.^43^ The phenotype was more severe in the recessive cases with early onset, and patients were wheelchair‐dependent by the time of diagnosis.^23^, ^33^, ^34^, ^37^, ^39^ Similar to other reports, intra and interfamilial phenotype variability were seen in African patients as the cases reported from Algeria.^36^, ^42^ Also, a case of CMT associated with hearing impairment was reported in an Algerian family.^44^ The disease course was slowly progressive in most of the cases, but a rapidly progressive case was found in an Algerian family.^44^ The demyelinating type was the most reported (Table 2). Nerve biopsy was performed in select cases and showed the classic aspect of “onion bulbs.”^34^, ^36^, ^40^, ^44^, ^45^ In addition, some axonal cases were reported with an important loss of large myelinating fibres and a few clusters of regeneration.^23^, ^47^ The main characteristics of the clinical profile are highlighted in Table 2.

**TABLE 2:** Main clinical and genetic characteristics of studies included in this review

Studies	Age of onset	Starting symptoms	Major neurological signs	Type of CMT	Genes	Variants	Methods of diagnosis
Aiyesimoju, 1984^30^	NR	NR	NR	NA	NA	NA	NP
LeGuern, 1996^67^	NR	NR	NR	ARCMT1	NI	NI	Homozygosity mapping
Kessali, 1997^44^	First decade	Foot and spine deformities	Distal muscle weakness in UL and LL, areflexia, foot deformities, kyphoscoliosis, hypoacusis and facial weakness	ARCMT	NI	NI	Linkage analysis
Meggouh, 1998^43^	Second decade	Distal LL muscle weakness and wasting	Distal muscle weakness predominantly in LL, tendon areflexia, pes cavus and kyphoscoliosis	CMTX	*Cx32/GJB1*	del499G	Sanger sequencing
Bouhouche, 1999^39^	Second decade	NR	Muscles weakness and wasting of the distal limbs, and areflexia predominantly in the lower limbs. Involvement of the proximal muscles in few patients. Pes cavus and severe kyphoscoliosis.	ARCMT2	NI	NI	Linkage analysis, physical mapping and direct sequencing
Othmane, 1999^16^	First/second decade	NR	Atrophy and weakness of intrinsic foot muscles, peronei, and anterior tibial muscles. Pes cavus and hammer toes.	CMT4B	NI	NI	Homozygosity mapping and linkage analysis
Barhoumi, 2001^38^	First decade	Walking difficulties	Severe distal muscle wasting, and atrophy of legs and of small muscles of hands. Steppage gait with bilateral foot drop, brisk tendon reflexes in UL and knee, and absent ankle reflexes. Distal sensory loss in LL including sense of touch, pain, proprioception and pallesthesia.	ARCMT2	NI	NI	Homozygosity mapping and linkage analysis
Baxter, 2001^33^	First decade	Muscle weakness	Weakness and atrophy of the feet and hands (clawhands). Wheelchair‐dependent and/or develop kyphosis. Mild sensory loss, proprioception and vibration senses.	CMT4	*GDAP1*	c.G92A; p.W31X	Direct sequencing
						c.G482A; p.R161H	
Sandre‐Giovannoli, 2002^68^	First decade	Muscle weakness	NR	ARCMT2	*LMNA*	c.C892T, p.R298C	Direct sequencing
Kakar, 2003^35^	Fifth decade	Bilateral numbness and tingling in feet	Severe atrophy and weakness of the distal arm and legs. Tendon areflexic, with flexor plantar responses. There was sensory loss of all modalities in a glove and stocking distribution. Gait was abnormal with bilateral foot drop. He had pes cavus.	CMT1B	*MPZ*	c.C234G, p.S78W	Direct sequencing
Azzedine, 2003^31^	First/second decade	Muscle weakness	Motor and sensory loss, areflexia, foot deformities and scoliosis	CMT4B2	*MTMR13*	c.C2875T, p.Gln956Stop^a^	Sanger sequencing
						c.C3586T; p.Arg1196Stop^a^	
Chaouch, 2003^45^	First/second/third decade	Muscle weakness	Weakness and amyotrophy of proximal muscles of pelvic girdle. Variable distal sensory disturbances with a glove and stock distribution.	ARCMT2	*LMNA*	c.C892T; p.R298C	Sanger sequencing
Birouk, 2003^23^	First decade	Foot deformities and muscle weakness	Distal muscle weakness and wasting of legs, predominantly in peroneal muscles, with severe foot deformities of the pes equinovarus type. Total areflexia, and loss of proprioception in the lower limbs.	ARCMT2	*GDAP1*	S194X	Sanger sequencing
Tazir, 2004^42^	First/second/third decade	Difficulty to running and walking	Distal and proximal muscle weakness, sensory loss, amyotrophy and areflexia. Foot deformities with pes cavus, scoliosis.	ARCMT2	*LMNA*	c.C892T, p.R298C	Sanger sequencing
Azzedine, 2006^69^	First decade	Scoliosis and kyphoscoliosis	Scoliosis or kyphoscoliosis and foot deformities	CMT4C	*SH3TC2*	del GCTGCTCGGAG; A74_77 indel fsX128^a^	Direct sequencing
						IVS10‐1G/A^a^	
						c. 2190delC; p.E731fsX750^a^	
						c.C 2710T; p.R904X^a^	
						c. C2860T; p.R954X^a^	
Bösenberg, 2006^48^	First/second decade	Running difficulty and peroneal spasm	Wasting of the thenar, eminence and the interossei of both hands and feet. Deep tendon reflexes were absent, slight sensory loss in his hands and feet. Feet deformities.	CMT1A	*PMP22*	*PMP22* duplication	NR
Onwuewe, 2007^50^	Second decade	Paraesthesia	Distal quadriparesis, spontaneous fasciculations, hyporeflexia and loss of proprioception	CMT1	NA	NA	NP
Bouhouche, 2007^40^	First decade	Hypotonia at birth and walking delay	Predominantly distal motor deficit and atrophy of both UL and LL. Atrophy and weakness of proximal muscles, distal sensory impairment involving particularly proprioception in the LL.	CMT4A	*GDAP1*	c.C233T; p. P78L	Linkage analysis and direct sequencing
Delague, 2007^70^	First decade	Delayed walking	Muscle weakness and amyotrophy in the distal extremities, marked feet abnormalities (pes cavus), absent tendon reflexes in the four limbs, ataxia, and a waddling gait	CMT4H	*FGD4/FRABIN*	c.T893C, p.Met298Thr	Direct sequencing
Bouhouche, 2007^40^	First decade	hypotonia at birth and delayed first motor acquisition	Distal muscle weakness, foot deformities and claw fingers, areflexia, sensory loss, wheelchair bound	CMT2B1	*LMNA*	c.892C>T; p.Arg298Cys^a^	Microsatellite markers and direct sequencing
				CMT4A	*GDAP1*	c.C581G; p.S194X^a^	
Hamadouche, 2008^49^	First/second/third decade	NR	NR	ARCMT2	*LMNA*	c.892C>T; p.Arg298Cys	Sanger sequencing
Nouioua, 2011^37^	First/second decade	Spine deformities and gait instability	Predominantly motor neuropathy with a steppage gait and distal limb weakness and wasting, claw hands and sensory loss, stridor and breathing difficulties	CMT4B1	*MTMR2*	c.331dupA; p.Arg111LysfsX24^a^	Sanger sequencing
				CMT4F	*PRX*	c.1090C>T; p.Arg364X^a^	
Baudot, 2012^32^	NR	NR	NR	CMT4H	*FGD4/FRABIN*	c.1325G>A; p.Arg442His	Sanger Sequencing
Kandil, 2012^29^	NR	NR	NR	NR	NA	NA	NP
Boubaker, 2013^34^	First decade	Gait disturbance	Amyotrophy and muscle weakness in the UL and LL. Muscle tone was low and deep tendon reflexes were absent. Walking on her tip toes, pes cavus and mild scoliosis.	CMT4H	*FGD4/FRABIN*	c.514_515insG; p.Ala172Glyfs*27	Sanger sequencing
Mathis, 2014^36^	First decade	Walking difficulties	Weak deep tendon reflexes in all four limbs and kyphoscoliosis	CMT1A	*PMP22*	*PMP22* duplication	MLPA and direct sequencing
Yalcouyé, 2019^7^	Second decade	UL muscle weakness	Distal muscle and sensory loss, muscle weakness and steppage gait	CMT2D	*GARS*	c.794C>A; Ser265Tyr	NGS (CMT gene panel)
Manyeruke, 2020^46^	First decade	NR	NR	CMT1A	*PMP22*	*PMP22* duplication	NR

Abbreviations: LL, lower limbers; MLPA, Multiplex Ligation Probe Amplification; NA, not applicable; NGS, next generation sequencing; NI, not identified; NP, not performed; NR, not reported; UL, upper limbs.

^a^ Different families.

### 3.3 Pedigrees' analysis

The pattern of inheritance was AR in most cases (91.2%, n = 97) (Figure 3G‐I) while AD represented 4.9%, and X‐linked and unknown patterns were seen in 3.9% each. Consanguinity was reported in 62% (n = 66) of families.^34^, ^37^

### 3.4 Genetic analysis

Several techniques were used to identify the causative genes associated with CMT over the time (Table 2). While the recent studies use NGS methods and targeted CMT genes panel, in the past decades, Multiplex Ligation Probe Amplification (MLPA), homozygosity mapping, and direct sequencing were used to identify the causative genes in CMT. The latter methods were mostly used in the studies reported here and allowed the identification of four genetic loci and 22 variants in African families, representing more than half of the cases (Table 2). However, the genes and the variants in the mapped loci were not identified. In a consanguineous family from Morocco, Othmane et al mapped the first CMT‐associated (CMT4B) locus (11p15) in Africa.^16^ Of note, none of the studies reviewed here used whole‐exome sequencing (WES) nor whole‐genome sequencing (WGS) to diagnose CMT cases.

To date, 11 genes including *LMNA*, *GDAP1*, *PMP22*, *MTMR2*, *MTMR13*, *Cx32/GJB1*, *PRX*, *MPZ*, *FGD4/FRABIN*, *SH3TC2*, and *GARS* have been associated with CMT in Africa. The most common genes were *LMNA*, *GDAP1*, and *SH3TC2* representing more than 80% (n = 65) of the molecularly diagnosed CMT cases in Africa.

The specific genes and the respective countries are shown in Figure 2. Variants in the *PMP22* gene were reported in four families only.^36^, ^46^, ^48^ Interestingly, a variant (c.C892T, p.R298C) in the *LMNA* gene was found to have a founder effect in North‐Western Africa (Algeria and Morocco).^49^ In other studies, most of the variants reported were novel^7^, ^32^, ^34^, ^35^, ^37^, ^43^ and no genes have been identified in a few studies.^16^, ^39^, ^44^, ^50^ This is not surprising since most of these studies were conducted more than two decades ago, and the techniques used were less efficient compared to NGS. NGS with a CMT gene panel testing was performed in only one study.^7^ All the genes and variants reported are summarised in Table 2.

## 4 DISCUSSION

To the best of our knowledge, this review is the most comprehensive and complete report on the epidemiological, clinical, and genetic features of CMT in Africa. It revealed the lack of data from most African countries, especially from SSA. The review has also allowed us to identify the genetic profile of CMT in Africa and suggests a difference from what is reported to date in the Western countries characterised by a lower contribution of *PMP22‐*associated variants in Africa, higher rate of novel and founder variants in known genes, likely related to higher consanguinity rates. In contrast to the high‐income countries, the prevalence or incidence of CMT in Africa is still largely unknown. Two clinical studies published in the 1980s have reported prevalence rates of 8/100 000 and 10/100 000 in Libya^51^ and in Nigeria,^52^ respectively. More recently, an estimated prevalence of 12/100 000 was reported in Egypt.^29^ Most of the studies included in this review were case reports on familial or isolated cases, illustrating a widely variable regional epidemiological description of CMT in Africa.^28^, ^47^

Similarly, the clinical description was similar to other reports worldwide. In most patients, symptoms appear during the first or second decade of life with an insidious onset and a slowly progressive weakness that starts in the lower extremities and later involves upper extremities.^53^, ^54^, ^55^ Diverse phenotypes were reported in Africa including asymptomatic, mild, moderate, and severe forms of CMT.^7^, ^23^, ^34^, ^37^, ^40^ These data confirm its clinical heterogeneity reported in other populations.^9^, ^55^ However, the clinical presentation seems more severe in the African families^39^ than reported elsewhere.^56^ This could be due to other genetic modifiers, environmental factors, the differences in the care, or the high frequency of the reported recessive cases of CMT, known to be more severe. The distribution of muscle weakness is mainly in the distal part but can also be proximal as reported in some studies.^45^, ^57^ In addition to muscle weakness, other motor signs include decreased or absent tendon reflexes, amyotrophy, and walking difficulties with steppage gait. Similar to other reports, some rare cases can cause respiratory failure or breathing difficulties like the case reported by Nouioua et al.^37^, ^58^, ^59^ Sensory impairment is typically associated with the phenotype, affecting generally the distal part in “gloves and socks” pattern.^57^, ^60^ The disease course is slowly progressive in most cases, but in exceptional cases, it can progress rapidly.^44^ In this study, most families segregated CMT disease in its recessive form associated with a high rate of consanguinity. This is different from the dominant manner inheritance pattern that is the most common reported worldwide,^42^ but could be due to the underreported cases in Africa.

The histological study has a role in identifying underlying genetic aetiology in sporadic cases, and it helps distinguishing CMT from acquired peripheral neuropathies.^57^ Nerve biopsy may also support a functional association when the genetic tests detect “variants of uncertain significance” or a novel variant.^57^ However, these were not performed as standard procedures in the studies reviewed here.^7^, ^30^, ^32^, ^36^, ^41^ Nerve conduction studies are an important step in the algorithm of CMT diagnosis, and allow the classification of different CMT types.^57^ This testing was performed in almost all the studies, though not all patients in each study were screened.^30^, ^50^

The present review highlighted some regional specificity with *LMNA* and *GDAP1* genes mostly found in North Africa where most of the studies were reported from, a region known for its high consanguinity rates as confirmed in this review.^61^ The consanguinity rate was also high in Mali, Morocco, Algeria, and Egypt,^23^, ^24^, ^25^, ^39^, ^40^, ^45^ a population profile that will favour genes discovery in the future. Indeed, the data also showed limited use of NGS to investigate CMT in Africa, and no study used WES or WGS. Indeed, WES/WGS are highly likely to identify novel genes and variants in known genes, particularly among understudied and highly genetically diverse populations of Africa. Despite that limited data were reported from Africa when compared to those from Europe and America, this study also confirms the genetic heterogeneity of CMT disease in Africa in line with the global knowledge, with >100 different genes identified to date.^9^, ^10^ All Mendelian models of inheritance were seen, but the dominant pattern is the most commonly reported worldwide^42^ while recessive cases were shown to be predominant in the cases described in Africa; likely associated with high consanguinity rate of most cases reported from North Africa.^49^ Most demyelinating CMT types result from mutations in genes expressed by SCs, whereas axonal types result from mutations expressed by neurons and their axons.^26^ Recent studies reported that the increasing insights into the molecular‐genetic mechanisms have revealed potential therapeutic targets.^26^ These will enable the development of novel therapeutics for genetic neuropathies that remain, in their majority, without effective treatment.^9^

The techniques used in Africa in the past were mostly MLPA, targeted sequencing, and homozygosity mapping. These have allowed the identification of genetic loci and known and novel variants for CMT cases. Despite the rapid evolvement of the genetic diagnosis of CMT in recent years with the advent of the NGS technology,^9^, ^60^ only one study in Africa has used it to diagnose a CMT case.^7^ NGS technology allows multiple parallel sequencing of either targeted genes, only the protein‐coding sequences (WES) or the whole genome (WGS).^9^, ^10^, ^60^ The challenge is how best to use these in clinical practice. To answer this question, Gonzaga‐Jauregui et al performed WES on individuals with CMT and reported a diagnostic rate of 45%.^62^ Recent studies have confirmed the efficiency of NGS in diagnosing CMT cases.^9^ In a cohort of pre‐excluded PMP22dup/del from Japan, authors identified the causative genes in 30% of the cases, and the most common genes were *GJB1*, *MFN2*, and *MPZ*.^55^ The overall diagnosis rate is higher in demyelinating CMT compared to the axonal type.^8^, ^55^

The molecular profile of CMT is sparse but globally *PMP22*, *GJB1*, *MFN2*, *MPZ* genes explain at least 90% of CMT cases.^3^, ^9^, ^10^ This epidemiology described above may not necessarily be extrapolated to other populations with different ethnic backgrounds, most notably those from the African continent which remains understudied^21^, ^63^ and is under‐represented in large population genetic databases.^64^ In fact, the genetic epidemiology profile from the studies reviewed here does not reflect what was reported in other populations. While CMT1A represents more than 60% of all CMTs,^9^ to date, the commonest CMT gene (*PMP22*) has been reported in only four families in Africa.^36^, ^46^, ^48^ Moreover, an AR CMT case (CMT4B) was first mapped in a family from Africa, before the gene was identified in subsequent studies done abroad^16^; suggesting that the African population harbours specific gene variants for CMT but the limited access to diagnosis tools may delay the molecular diagnosis confirmation.

The scarcity of the most common CMT genes in Africa might be associated with the limited number of studies as it is expected that the prevalence of CMT1A (and HNPP) could be similar in populations worldwide. It is possible CMT1A might be under reported because the phenotype is already well known, and only the most severely affected patients come to the medical attention in many African regions, owing to the limited access to diagnostic tools, and to scarce neurology specialists. It is also possible that the findings of the current review may be due to the genetic diversity of African populations, the population structure, the consanguinity rates, or the genetic drift. Therefore, this stresses the need for more studies on the genetics of CMT in Africa using NGS, with the potential of uncovering novel genes or variants important for the function of the peripheral nerve system. CMT is a disabling condition that does not have a cure, but the advances in the understanding of its pathophysiology have advanced research in the identification of therapeutic targets in human and animal models.^11^, ^55^, ^65^, ^66^ The extension of such studies to Africa could be especially beneficial and equitable.

## 5 STRENGTHS AND LIMITATIONS

To the best of our knowledge, this review provides the most comprehensive and complete data on CMT in Africa. It summarised the available data on the epidemiological, clinical, and genetic profiles of CMT in Africa. It identified the enormous gaps in the knowledge of CMT in Africa compared to developed countries and highlighted the necessity to undertake large‐scale genetic studies on CMT in Africa to further our understanding of its global epidemiology and perhaps identify other therapeutic perspectives. Therefore, this review may be the first step for future perspectives in the research of CMT in Africa. However, this study has some limitations. First, the absence of nationwide studies in Africa, and most of the studies included herein were case reports which are obviously limited with regards to epidemiological data. Second, the keywords we used for searches may have missed some articles that do not include those words. Third, the language restriction to English and French may have also missed some articles reported in other languages. Fourth, many African researchers do not have access to indexed journals and may have published in journals that our selection criteria do not catch.

## 6 CONCLUSION

This study reveals that CMT is not rare, and likely underreported in Africa and describes the current clinical and genetic profile. Large and multicentric cohort studies in Africa would not only inform the genetic epidemiology of CMT in this region but could also lead to new discoveries important to the global research effort for therapeutic perspectives. The increasing access to NGS technologies offers to African scientists a unique opportunity to fully describe relevant variants in known genes and to discover novel CMT‐associated genes that may improve our understanding and care of this condition in Africa.

## CONFLICT OF INTEREST

The authors declare no conflicts of interest.

## AUTHOR CONTRIBUTIONS

**Abdoulaye Yalcouyé**: Developed the methodology; database search; analysed and interpreted the data, wrote the first draft; read and agreed to the published version of the manuscript. **Kevin Esoh**: Developed the methodology; critically revised successive drafts of the manuscript; read and agreed to the published version of the manuscript **Guida Landouré**: Conceived the study; critically revised successive drafts of the manuscript; supervised the project; read and agreed to the published version of the manuscript **Ambroise Wonkam**: Conceived the study; critically revised successive drafts of the manuscript; supervised the project; read and agreed to the published version of the manuscript.

## Data Availability

The data that support the findings of this study are available from the corresponding author upon reasonable request.
